# Impact of Pituitary Stalk Preservation on Tumor Recurrence/Progression and Surgically Induced Endocrinopathy After Endoscopic Endonasal Resection of Suprasellar Craniopharyngiomas

**DOI:** 10.3389/fneur.2021.753944

**Published:** 2021-11-04

**Authors:** Zhengyuan Chen, Zengyi Ma, Wenqiang He, Xuefei Shou, Zhao Ye, Yichao Zhang, Qilin Zhang, Nidan Qiao, Xiang Zhou, Xiaoyun Cao, Min He, Zhaoyun Zhang, Hongying Ye, Yiming Li, Shiqi Li, Yao Zhao, Ming Shen, Yongfei Wang

**Affiliations:** ^1^Department of Neurosurgery, Huashan Hospital, Shanghai Medical College, Fudan University, Shanghai, China; ^2^Neurosurgical Institute of Fudan University, Shanghai, China; ^3^Shanghai Clinical Medical Center of Neurosurgery, Shanghai, China; ^4^Shanghai Key Laboratory of Brain Function Restoration and Neural Regeneration, Shanghai, China; ^5^Shanghai Pituitary Tumor Center, Shanghai, China; ^6^National Center for Neurological Disorder, Shanghai, China; ^7^Department of Endocrinology and Metabolism, Huashan Hospital, Shanghai Medical College, Fudan University, Shanghai, China; ^8^Research Units of New Technologies of Micro-Endoscopy Combination in Skull Base Surgery (2018RU008), Chinese Academy of Medical Sciences, Beijing, China

**Keywords:** craniopharyngioma, EEA surgery, pituitary stalk, recurrence, progression, endocrinopathy

## Abstract

**Objective:** To investigate the factors associated with recurrence/progression after endoscopic endonasal resection of suprasellar craniopharyngiomas. Special attention was paid to assess the impact of pituitary stalk preservation on tumor recurrence/progression and endocrinological outcomes.

**Methods:** We retrospectively recruited 73 patients with suprasellar craniopharyngiomas undergone endoscopic endonasal approach (EEA) surgery from September 2014 to May 2019 and assessed their clinical characteristics, surgical outcomes, and recurrence/progression. Stalk preservation or sacrifice was determined by reviewing operative records, videos, and post-operative magnetic resonance imaging.

**Results:** Gross total resection (GTR) was achieved in 51 cases (69.9%). Tumor recurrence was seen in 5 cases (9.8%) and progression was seen in 8 cases (36.4%), respectively. GTR (OR = 0.248 CI 0.081–0.759; *p* = 0.015) was the only independent factor influencing recurrence/progression. Kaplan-Meier survival analysis showed that the mean recurrence/progression-free survival were 53 (95% CI 48–59) and 39 (95% CI 28–50) months, respectively, in patients with and without GTR (*p* = 0.011). Pituitary stalk preservation was more common in cases with peripheral type tumors (83% vs. 30%, *p* < 0.01). Preserving the pituitary stalk does not appear to decrease the percentage of GTR (75.5% vs. 55.0%, *p* = 0.089), or increase the rate of tumor recurrence (12.5% vs. 0%, *p* = 0.508) or progression (46.2% vs. 22.2%, *p* = 0.486). However, surgically induced hypothyroidism (60.5% vs. 100%, *p* = 0.041) and diabetes insipidus (35.1% vs. 81.8%, *p* = 0.017) were significantly lower in patients with stalk preservation. For patients who had hypopituitarism before EEA, there was no difference between those with and without stalk preservation regarding post-operative hypopituitarism (*p* > 0.05).

**Conclusion:** GTR is the only independent predictor of recurrence/progression after EEA surgery for suprasellar craniopharyngiomas. Preserving the pituitary stalk does not appear to increase the risk of non-GTR and tumor recurrence/progression and might help reduce the risk of surgically induced hypothyroidism and diabetes insipidus. We recommend preserving the pituitary stalk in peripheral type suprasellar craniopharyngiomas with normal pituitary function, especially in cases without hypothyroidism or diabetes insipidus. On the other hand, stalk sacrifice could be considered in central type tumors with severe pre-operative endocrinopathy.

## Introduction

Craniopharyngioma is a common congenital tumor that constitutes about 2–6% of primary intracranial tumors, leading to visual disturbance, endocrine dysfunction, or cranial nerve palsy (1). Given its benign nature, craniopharyngioma is quite challenging due to its anatomical proximity to vital structures, including hypothalamic-pituitary axes, optical apparatus, and cranial nerves (2). It is ideal if gross total resection (GTR) with preservation of the hypothalamic function could be achieved (3). Otherwise, adjuvant radiotherapy is reserved in selected cases of residual or recurrent tumors (4).

Due to their origin from remnants of the craniopharyngeal duct epithelium, craniopharyngiomas may arise anywhere along the pituitary-hypothalamic axis. The pituitary stalk plays a crucial role in hypothalamic-pituitary functioning and is a vital structure during the surgical resection of suprasellar craniopharyngiomas. Damage to the pituitary stalk may lead to endocrine dysfunction, disruption of the water-electrolyte balance, central diabetes insipidus (DI), and other clinical manifestations (5). However, preserving the pituitary stalk might increase the risk of recurrence. Therefore, the choice of preserving or sacrificing the pituitary stalk is of great significance during surgical resection of suprasellar craniopharyngiomas. However, to the best of our knowledge, it still has controversies in literature (6). Several studies recommended preserving the pituitary stalk as much as possible, as the recurrence rate is not affected but might yield improved endocrinological outcomes (7–9). By contrast, some researchers found a weak association between stalk preservation and post-operative endocrinological benefits (10, 11). Moreover, stalk-preservation without achieving GTR will inevitably put patients at the risk of tumor recurrence.

Compared with transcranial approaches, the endoscopic endonasal approach (EEA) provides an ideal visualization of the ventral skull base, enabling early identification of the pituitary stalk (12, 13). Up to now, data reflecting the association between stalk preservation/sacrifice and post-operative recurrence and endocrinological outcomes in EEA is limited (14, 15). Does preserving the pituitary stalk lead to a higher risk of recurrence or progression? Does it contribute to a more favorable pituitary function? In the present study, we addressed these issues by evaluating the outcomes of EEA in 73 suprasellar craniopharyngiomas with a median follow-up time of 19 months.

## Materials and Methods

### Patient Enrollment

We retrospectively collected data from patients treated in Huashan Hospital, the largest tertiary referral center in East China, between September 2014 and May 2019. The inclusion criteria were: (i) pathologically confirmed craniopharyngiomas; (ii) suprasellar type tumors based on MRI and intraoperative observation; (iii) treated by EEA. Patients who were followed up <6 months and those who were managed by prophylactic radiotherapy after surgery without evidence of recurrence/progression were excluded. The patients' medical records and imaging studies were reviewed after obtaining approval from the Huashan Institutional Review Board.

### Endocrinological Evaluation

All patients were subject to endocrinological evaluation at baseline as well as post-operative follow-ups to screen for endocrinopathy. Morning free cortisol, adrenocorticotropin (ACTH), thyroid-stimulating hormone (TSH), total triiodothyronine (TT3) and thyroxine (TT4), free triiodothyronine (FT3) and thyroxine (FT4), prolactin, testosterone, estradiol, progesterone, luteinizing hormone (LH), and follicle-stimulating hormone (FSH) were tested. Hypopituitarism was diagnosed if hormones were lower than the normal range for each axis and not adequately responded to the stimulation test or already underwent hormone replacement therapy. The diagnosis of DI was based on thirst, urine output, serum electrolyte levels, urine specific gravity, and serum/urine osmolarity.

### Neuroimaging

All patients underwent spin-echo sequence T1 post-contrast enhanced sagittal and coronal MRI pre-operatively, using a 3.0-T whole-body scanner (General Electric Medical Systems, 118 Milwaukee, MI). Certain patients had also undergone CT angiography. Tumor location, size, calcification, and cyst formation, were recorded. Tumors were classified into the central and peripheral types based on their relationship with the pituitary stalk (16). Central type refers to tumors that grow within the stalk, and no definite origin site can be identified. Peripheral type tumor arises from the stalk, extending laterally in an exophytic pattern, and the residual stalk is usually displaced. The resection degree was determined by reviewing post-operative MRI within 72 h after surgery. A GTR was achieved when complete removal of the tumor had been carried out, subtotal resection (STR) was achieved when more than 75% of the tumor was removed, and partial resection (PR) was achieved when <75% of the tumor was removed. Post-operative MRI was performed 3 months post-operatively and then at semi-annual or annual intervals during follow-up. Recurrence was defined as newly discovered neoplasms after GTR. Progression was defined as enlarged residual tumors after non-GTR.

### Surgical Procedure, Follow-Up, and Adjuvant Therapy

All patients underwent an extended endoscopic endonasal trans-tuberculum approach. After sphenoidotomy and partial posterior ethmoidectomy, a wide exposure of the sellar and suprasellar area was achieved. The bone covering the sella turcica, the tuberculum, and the chiasmatic sulcus were widely opened to facilitate surgical freedom during suprasellar manipulations. Ligation of the superior intercavernous sinus was performed while opening the skull base dura. After opening the arachnoid membrane and releasing cerebrospinal fluid (CSF), the pituitary stalk could be recognized in most cases. Intracapsular debulking was carried out, followed by extracapsular dissection. We cut the tumor beside the pituitary stalk for peripheral type craniopharyngiomas and preserve the stalk as much as possible. However, if preserving the stalk hindered GTR, we sacrificed it. We vertically cut open the stalk for central type tumors, remove the tumor inside, and see if we could preserve some normal stalk tissues connecting the hypothalamus. However, there were no preservable stalk tissues in most central-type cases. We dissected tumors from the hypothalamus along the gliosis between them. If it was too adherent, this part of the tumor was reserved. The skull base reconstruction was performed using the standard multilayer technique, including a pedicled nasal septal flap.

All patients were closely followed up immediately and 1, 3, 6 months, and semi-annually or annually after surgery. Adjuvant radiotherapy was adopted for patients with recurrence after GTR or progression after non-GTR.

### Statistical Analysis

Statistical analysis was performed using SPSS 22.0, and data were presented as mean ± SD (or median with interquartile range) for continuous variables normally (or not normally) distributed and as the frequency for categorical variables. Normality was tested using the Kolmogorov-Smirnov test. Means were compared using the unpaired *t*-test when data distribution was normal or by the Wilcoxon rank-sum (Mann-Whitney) test when variables were not normally distributed. For categorical variables, differences were analyzed by the Chi-square test or Fisher's exact-tests as appropriate. Cox regression analysis was used to determine the potential risk factors for predicting recurrence/progression. Recurrence/progression-free survival was estimated using the Kaplan-Meier method, and Kaplan-Meier survival curves were generated. A two-tailed *P*-value < 0.05 was considered significant.

## Results

### Patients Characteristics

The characteristic of 73 patients with suprasellar craniopharyngiomas who underwent EEA surgery was summarized in Table 1. There were 38 males and 35 females with a mean age of 39 ± 16 years. Nine patients (12.3%) had a history of surgery, and five patients (6.8%) had a history of fractionated radiotherapy or stereotactic radiosurgery, respectively. Forty-one patients complained of visual deterioration, 22 of headache, 19 of amenorrhea, 13 of drowsiness, 11 of polyuria/polydipsia, 5 of vomiting, 4 of developmental retardation, and one of obesity. There were 23 (31.5%) central type tumors and 50 (68.5%) peripheral type tumors. The percentage of pre-operative hypopituitarism was 32.9% of hypoadrenalism, 35.6% of hypothyroidism, 67.1% of hypogonadism and 34.2% of DI, respectively.

**Table 1:** Clinical characteristics of 73 patients with suprasellar craniopharyngiomas.

**Characteristics**	**Value**
**Demographic features**	
Gender (male)	38 (52.1%)
Age at diagnosis (years)	39 (16)
Follow-up time (months)	19 [12–36]
**Previous treatment history**	
Surgery	9 (12.3%)
Radiotherapy	5 (6.8%)
**Clinical manifestation**	
Visual impairment	41 (56.2%)
Headache	22 (30.1%)
Amenorrhea	19 (26.0%)
Drowsiness	13 (17.8%)
Polyuria/polydipsia	11 (15.1%)
Vomiting	5 (6.8%)
Developmental retardation	4 (5.5%)
Obesity	1 (1.4%)
**Imaging and endocrinological features**	
Maximum diameter (cm)	3.0 [2.0–3.8]
Tumor type	
Central type	23 (31.5%)
Peripheral type	50 (68.5%)
Pre-operative endocrinopathy	
Hypoadrenalism	24 (32.9%)
Hypothyroidism	26 (35.6%)
Hypogonadism	49 (67.1%)
Diabetes insipidus	25 (34.2%)
**Treatment features**	
Resection degree	
Gross total resection	51 (69.9%)
Subtotal resection	16 (21.9%)
Partial resection	6 (8.2%)
Pituitary stalk preservation	53 (72.6%)

*Continuous variables with normal distribution were displayed as mean (standard deviation), continuous variables without normal distribution were displayed as median [interquartile range], categorical variables were displayed as count (proportion)*.

### Surgical Outcomes and Complications

GTR, STR, and PR were achieved in 51 (69.8%), 16 (21.9%), and 6 (8.2%) patients, respectively. The pituitary stalk was morphologically preserved in 53 (72.6%) out of 73 patients. None of the patients died during the study period. All of them experienced intraoperative CSF leakage due to the extended approach. One (1.4%) patient experienced meningitis and was cured by antibiotics. Two (2.7%) patients had epistaxis. No incidents of intraoperative carotid injury, post-operative CSF leakage, or intracerebral hemorrhage occurred. Patients were followed up for 6–60 months with a median time of 19 months. At the last investigation, tumor recurrence was seen in 5 cases (9.8%) at 6, 12, 18, 20, and 24 months, respectively, after GTR. Tumor progression was seen in 8 cases (36.4%) at 6, 7, 8, 9, 18, 18, 24, and 24 months, respectively, after non-GTR. Six and seven patients with recurrence/progression were treated with reoperation and radiotherapy, respectively. For patients with normal pituitary function pre-operatively, surgically induced hypoadrenalism, hypothyroidism, hypogonadism, and DI developed in 55.1%, 68.1%, 37.5%, and 45.8% of them, respectively.

### Predictors of Recurrence/Progression

**Figure 1:**
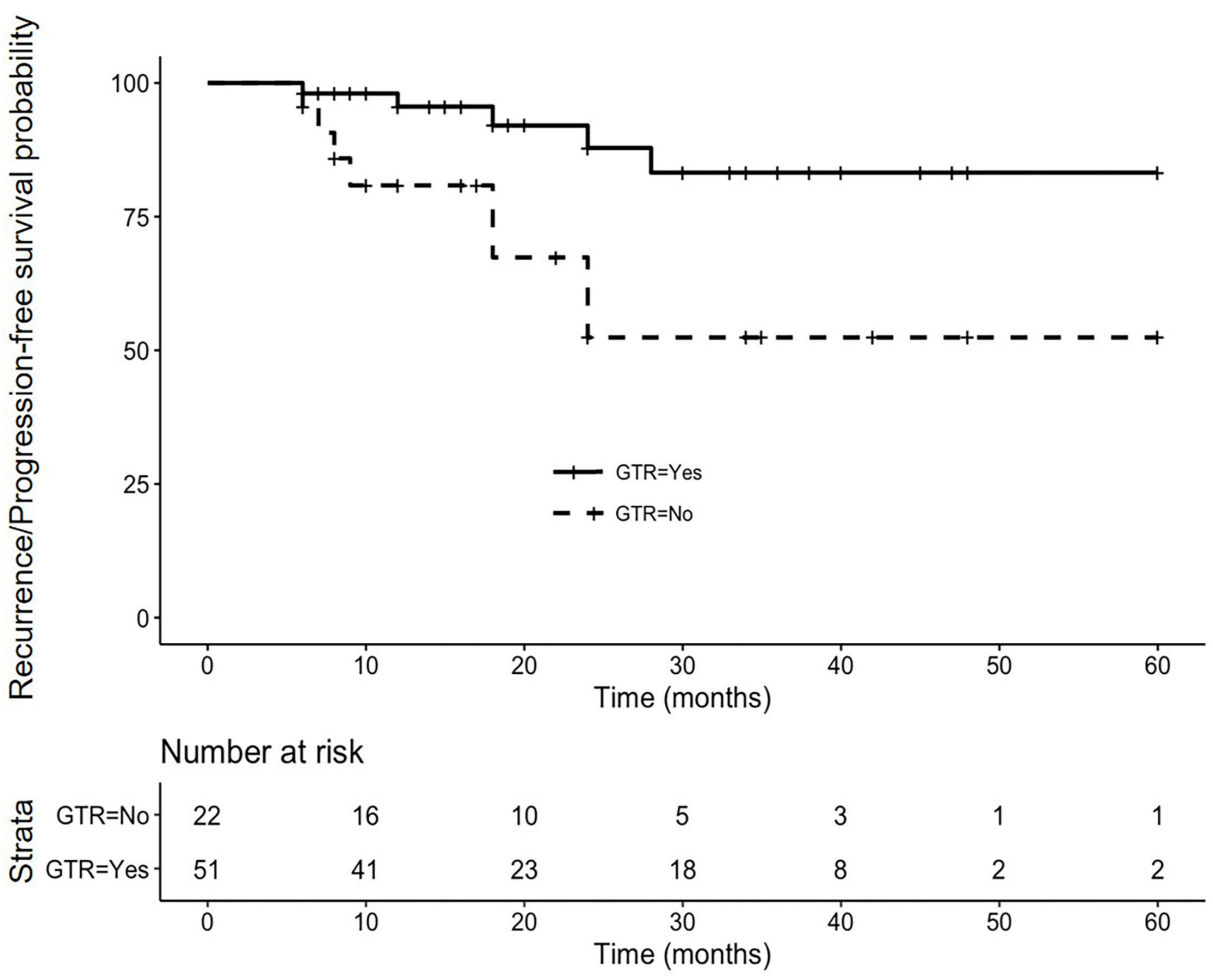
Cumulative recurrence/progression-free Kaplan-Meier curve in 73 suprasellar craniopharyngiomas underwent endoscopic endonasal surgeries. The mean recurrence/progression-free survival time was 53 (95% CI 48–59) and 39 (95% CI 28–50) months, respectively, in patients with (the solid line) and without (the dotted line) gross total resection (*p* = 0.011).

Patients were divided into the recurrence/progression (*n* = 13) and recurrence/progression-free (*n* = 60) groups (Table 2). Compared to recurrence/progression-free cases, those who presented with recurrence/progression were of significantly higher percentage of peripheral type tumors (100% vs. 61.7%, *p* = 0.018) and lower percentage of GTR (38.5% vs. 76.7%, *p* = 0.017). No differences were found concerning age, gender, follow-up time, previous treatment history, tumor size, pre-operative endocrinopathy, pituitary stalk preservation, and recurrence/progression-free survival time between the two groups (*p* > 0.05). Cox regression analysis showed that GTR (OR = 0.248 CI 0.081–0.759; *p* = 0.015) was the only independent factor influencing recurrence/progression (Table 3). Kaplan-Meier survival analysis showed that the mean recurrence/progression-free survival was 53 (95% CI 48–59) and 39 (95% CI 28–50) months, respectively, in patients with and without GTR (*p* = 0.011; Figure 1).

**Table 2:** Comparison between patients with recurrence/progression and patients without.

**Variable**	**Recurrence/progression group**	**Recurrence/progression-free group**	***p***
**Demographic features**			
Patients (*n*)	13 (17.8%)	60 (82.2%)	-
Gender (male)	5 (38.5%)	33 (55.0%)	0.279
Age at diagnosis (years)	41 (11)	39 (16)	0.616
Follow-up time (months)	21 (12)	19 [12–36]	0.634
**Previous treatment history**			
Surgery	2 (15.4%)	7 (11.7%)	1.000
Radiotherapy	2 (15.4%)	3 (5.0%)	0.214
**Imaging and endocrinological features**			
Maximum diameter (cm)	2.8 (0.8)	3.0 [2.0–3.9]	0.865
Tumor type			0.018^*^
Central type	0 (0%)	23 (38.3%)	
Peripheral type	13 (100%)	37 (61.7%)	
Pre-operative endocrinopathy			
Hypoadrenalism	3 (23.1%)	21 (35.0%)	0.614
Hypothyroidism	6 (46.2%)	20 (33.3%)	0.578
Hypogonadism	9 (69.2%)	40 (66.7%)	1.000
Diabetes insipidus	5 (38.5%)	20 (33.3%)	0.975
**Treatment features**			
Gross total resection	5 (38.5%)	46 (76.7%)	0.017^*^
Pituitary stalk preservation	11 (84.6%)	42 (70.0%)	0.466
Recurrence/progression-free survival time (months)	15 (7)	19 [12–36]	0.074

*Continuous variables with normal distribution were displayed as mean (standard deviation), continuous variables without normal distribution were displayed as median [interquartile range], categorical variables were displayed as count (proportion)*.

* *P < 0.05*.

**Table 3:** Cox regression analysis for predictors of recurrence/progression after surgery.

**Feature**	**OR (95% CI)**	***p***
Gross total resection	0.248 (0.081–0.759)	0.015^*^
Peripheral type tumor	3.270 × 10^5^ (0.000–4.23 × 10^195^)	0.955

*OR, odds ratio; CI, confidence interval*.

* *P < 0.05*.

### Factors Related to Pituitary Stalk Preservation During Surgery

Patients were divided into the stalk-preserved (*n* = 53) and stalk-sacrificed (*n* = 20) groups (Table 4). Compared to the stalk-sacrificed group, the stalk-preserved group had higher percentage of peripheral type tumors (83.0% vs. 30.0%, *p* < 0.001), lower percentage of pre-operative hypoadrenalism (22.6% vs. 60.0%, *p* = 0.002), and hypothyroidism (28.3 vs. 55.0%, *p* = 0.034). There were no significant differences regarding gender, age, previous treatment history, tumor size, pre-operative hypogonadism, and DI between the two groups (*p* > 0.05). After multivariable logistic regression analysis (Table 5), stalk preservation was found to be more common in peripheral type tumors (OR = 10.505 CI 2.968–37.176; *p* < 0.001) and less common in cases with pre-operative hypoadrenalism (OR = 0.220 CI 0.062–0.786; *p* = 0.020).

**Table 4:** Clinical characteristics between patients with pituitary stalk preservation and sacrifice.

**Variable**	**Stalk-preserved group**	**Stalk-sacrificed group**	***p***
**Demographic features**			
Patients (*n*)	53 (72.6%)	20 (27.4%)	-
Gender (male)	28 (52.8%)	10 (50.0%)	0.829
Age at diagnosis (years)	40 (16)	38 (15)	0.584
**Previous treatment history**			
Surgery	4 (7.5%)	5 (25.0%)	0.104
Radiotherapy	2 (3.8%)	3 (15.0%)	0.240
**Imaging and endocrinological features**			
Maximum diameter (cm)	3.0 [2.0–4.0]	2.8 (0.9)	0.879
Tumor type			0.000^*^
Central type	9 (17.0%)	14 (70.0%)	
Peripheral type	44 (83.0%)	6 (30.0%)	
Pre-operative endocrinopathy			
Hypoadrenalism	12 (22.6%)	12 (60.0%)	0.002^*^
Hypothyroidism	15 (28.3%)	11 (55.0%)	0.034^*^
Hypogonadism	32 (60.4%)	17 (85.0%)	0.086
Diabetes insipidus	16 (30.2%)	9 (45.0%)	0.234
**Treatment features**			
Gross total resection	40 (75.5%)	11 (55.0%)	0.089
Recurrence after gross total resection	5 (12.5%)	0 (0%)	0.508
Progression after non-gross total resection	6 (46.2%)	2 (22.2%)	0.486

*Continuous variables with normal distribution were displayed as mean (standard deviation), continuous variables without normal distribution were displayed as median [interquartile range], categorical variables were displayed as count (proportion)*.

* *P < 0.05*.

**Table 5:** Multivariable logistic regression analysis of factors related to pituitary stalk preservation.

**Feature**	**OR (95% CI)**	***p***
Peripheral type tumor	10.505 (2.968–37.176)	0.000^*^
Pre-operative hypoadrenalism	0.220 (0.062–0.786)	0.020^*^

*OR, odds ratio; CI, confidence interval*.

* *P < 0.05*.

### Impact of Pituitary Stalk Preservation on GTR, Tumor Recurrence/Progression

In the stalk-preserved group, there were 40 (75.5%), and 13 (24.5%) cases achieved GTR and non-GTR, respectively. In the stalk-sacrificed group, 11 (55.0%) and 9 (45.0%) cases achieved GTR and non-GTR, respectively. Preserving the pituitary stalk does not appear to decrease the percentage of GTR (75.5% vs. 55.0%, *P* = 0.089), or increase the rate of tumor recurrence (12.5% vs. 0%, *P* = 0.508) or progression (46.2% vs. 22.2%, *P* = 0.486) (Table 4). Multivariable logistic regression analysis provided similar results after adjusting confounding factors (*p* = 0.050 and 0.595 for GTR and recurrence/progression, respectively).

### Impact of Pituitary Stalk Preservation on Surgically Induced Endocrinopathy

At the last investigation, the overall percentage of hypopituitarism was 67.1% of hypoadrenalism, 76.7% of hypothyroidism, 77.0% of hypogonadism, and 58.9% of DI, which was higher than pre-operative status. Only individual cases experienced improved post-operative endocrine outcomes during follow-up.

Compared to the stalk-sacrificed group, post-operative endocrine outcomes were more favorable regarding hypoadrenalism (58.5% vs. 90.0%, *p* = 0.023), hypothyroidism (69.8% vs. 95%, *p* = 0.05), hypogonadism (68.2% vs. 100%, *p* = 0.006), and DI (49.1% vs. 85.0%, *p* = 0.012) in the stalk-preserved group.

**Figure 2:**
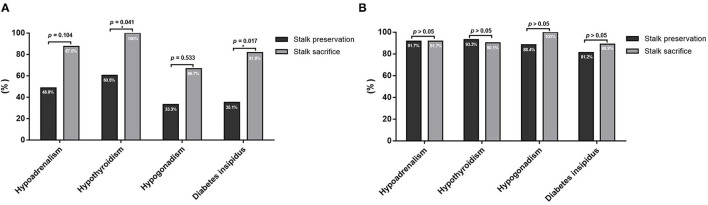
Comparison of post-operative hypopituitarism between patients with pituitary stalk preservation and sacrifice. **(A)** For patients with normal pituitary function before surgery, stalk preservation significantly decreased the percentage of surgically induced new-onset hypothyroidism and diabetes insipidus. **(B)** For patients who already had pre-operative hypopituitarism before EEA, there were no significant differences in the rate of post-operative hypopituitarism between patients with pituitary stalk preservation and sacrifice. *P < 0.05.

Furthermore, surgically induced new-onset hypothyroidism (60.5% vs. 100%, *p* = 0.041) and DI (35.1% vs. 81.8%, *p* = 0.017) were significantly lower in patients with stalk preservation (Figure 2A). Notably, for 15 patients with normal pituitary function, 8 (53.3%) of them maintained uncompromised pituitary function after stalk preservation.

In contrast, for patients who already had hypopituitarism before EEA, there was no significant difference between patients with and without stalk preservation regarding post-operative hypoadrenalism (91.7% vs. 91.7%, *p* = 1.000), hypothyroidism (93.3% vs. 90.1%, *p* = 1.000), hypogonadism (88.4% vs. 100%, *p* = 0.287), and DI (81.2% vs. 88.9%, *p* = 1.000) (Figure 2B).

## Discussion

The pituitary stalk connects the pituitary gland with the hypothalamus, constitutes an important anatomical basis for maintaining the hypothalamic-pituitary function. Suprasellar craniopharyngiomas originate from the pituitary stalk. Damage to the stalk, either pathologically or iatrogenically, compromises the endocrinological function and seriously affects patients' quality of life. However, preserving the pituitary stalk during surgery might shield the seed of the tumor, resulting in a higher risk of recurrence. From a study conducted by Xiao et al., the ultra-electron microscope was used to determine whether pituitary stalk specimens were invaded with tumor cells. The results revealed that all pituitary stalk samples (15/15, 100%) showed tumor invasion (9). Furthermore, a morphologically preserved pituitary stalk might not be functional. So, whether to sacrifice or preserve the stalk is still an issue of importance facing neurosurgeons.

Currently, two contradictory opinions existed in the intraoperative management strategy of the pituitary stalk. Honegger et al.'s series of 92 transcranial cases demonstrated that the post-operative endocrine status was generally better in patients with stalk preservation (7). The authors also recommended preserving the stalk as much as possible because it didn't seem to increase the risk of recurrence. By contrast, Jung et al. treated 17 pediatric craniopharyngiomas *via* transcranial approaches and found that preserving the pituitary stalk did not benefit post-operative endocrinological outcomes but significantly increased the risk of recurrence (10). Also, Xiao et al. recommended total resection of the tumor along with the pituitary stalk if the stalk was intraoperatively invaded (9). Sometimes, it is hard to say whether the pituitary stalk is preserved due to its deep location and the limited exposure of microscopic view during transcranial approaches. We might experience a “missing” pituitary stalk, but it is compressed to a corner that is difficult to observe under the microscope. On the contrary, we might think the pituitary stalk is preserved, but it has been transected at some site out of the surgical view. This makes one of the explanations of the different results mentioned above.

Compared with microscopic transcranial approaches, the EEA provides an ideal visualization of the ventral skull base, enabling early identification of the pituitary stalk (12, 13). The EEA is a perfect model to study the impact of pituitary stalk management on surgical and endocrinological outcomes of suprasellar craniopharyngiomas because the judgment of stalk preservation vs. sacrifice is very reliable in most cases. In a study conducted by Ordonez-Rubiano et al., the authors reported that stalk preservation during EEA reduced the rate of post-operative endocrinopathy but compromised GTR achievement with a higher rate of tumor recurrence or residual tumor regrowth (14). The authors recommended that stalk preservation should be considered if GTR could be achieved. In the present study, we demonstrated that GTR (OR = 0.248 CI 0.081–0.759; *p* = 0.015) was the only independent factor influencing recurrence/progression. We found a four-fold higher possibility of recurrence/progression in patients with non-GTR. Furthermore, preserving the pituitary stalk does not appear to decrease the percentage of GTR (75.5% vs. 55.0%, *p* = 0.089), or increase the rate of tumor recurrence (12.5% vs. 0%, *p* = 0.508) or progression (46.2% vs. 22.2%, *p* = 0.486). However, surgically induced hypothyroidism (60.5% vs. 100%, *p* = 0.041) and DI (35.1% vs. 81.8%, *p* = 0.017) were significantly lower in patients with stalk preservation. Most of our results were comparable to Ordonez-Rubiano et al.'s study. However, we didn't find stalk preservation decreased the rate of GTR. One explanation is that stalk preservation was found to be more common in peripheral type tumors in our study, which made GTR easier to achieve than in cases with central type tumors.

Regarding the endocrinological outcomes, the present study revealed a large proportion of post-operative hypopituitarism after EEA, even in patients with stalk preservation, which is similar to previous studies (17, 18). Patients should be informed and agreed with that. Attempt to preserve the stalk is time-consuming, but it is rewarded with improved post-operative pituitary function. However, for patients who already had severe hypopituitarism before EEA, stalk preservation didn't improve post-operative endocrinological outcomes. We recommend preserving the pituitary stalk as much as possible in peripheral type suprasellar craniopharyngiomas with normal pituitary function, especially in cases without hypothyroidism or DI. On the other hand, stalk sacrifice could be considered in central type tumors with severe pre-operative endocrinopathy.

Interestingly, we found there was one patient (5.0%) recovered adrenal function, one patient (5.0%) recovered thyroidal function, and one patient (5.0%) recovered posterior pituitary function even after stalk sacrifice. Ogawa and Nishizawa et al. also noticed that endocrinopathy could be partially reversible even with stalk sacrifice (11, 19). Further researches are needed to address this issue.

There are several limitations to our study. Firstly, because of the study's retrospective nature, the conclusion we draw may not be solid enough, and a prospective observational study is needed to strengthen our opinion further. Secondly, a limitation of comparison between studies is that not the same tumor location classification was used, and we had a relatively short follow-up period to estimate the long-term outcomes. We speculated that the short follow-up period might be related to the fact that ~80% of our patients come from other cities outside of Shanghai. It is difficult for everyone to visit us after 1–2 years of stable disease after surgery.

## Conclusion

GTR is the only independent predictor of recurrence/progression after EEA surgery for suprasellar craniopharyngiomas. Preserving the pituitary stalk does not appear to increase the risk of non-GTR and tumor recurrence/progression and might help reduce the risk of surgically induced hypothyroidism and DI.

## Data Availability Statement

The original contributions presented in the study are included in the article/Supplementary Material, further inquiries can be directed to the corresponding authors.

## Ethics Statement

The studies involving human participants were reviewed and approved by Ethics Committee, Huashan Hospital, Fudan University. Written informed consent to participate in this study was provided by the participants' legal guardian/next of kin.

## Author Contributions

ZC, ZM, MS, and YW: study design, implementation and supervision, and draft organization. YW and YZhao: surgical procedure. MS, WH, ZY, XZ, XS, XC, SL, and YZhan: patient recruitment. MH, ZZ, HY, and YL: endocrinological evaluation. ZM and QZ: radiological examination. NQ and ZM: statistical analysis. ZC and MS: clinical data collection. All authors draft final review.

## Funding

This work was supported by the National Natural Science Foundation of China (81602191), received by MS; the National Project in promoting the diagnosis and treatment of major diseases by MDT, received by YZhao; and the CAMS Innovation Fund for Medical Sciences (CIFMS, 2019-I2M-5-008), received by YW. The funders had no role in the study design, data collection and analysis, decision to publish, or preparation of the manuscript.

## Conflict of Interest

The authors declare that the research was conducted in the absence of any commercial or financial relationships that could be construed as a potential conflict of interest.

## Publisher's Note

All claims expressed in this article are solely those of the authors and do not necessarily represent those of their affiliated organizations, or those of the publisher, the editors and the reviewers. Any product that may be evaluated in this article, or claim that may be made by its manufacturer, is not guaranteed or endorsed by the publisher.
