# Supplementary figures and images for: Impact of Pituitary Stalk Preservation on Tumor Recurrence/Progression and Surgically Induced Endocrinopathy After Endoscopic Endonasal Resection of Suprasellar Craniopharyngiomas
Source: Front Neurol. 2021 Nov 4;12:753944. doi: 10.3389/fneur.2021.753944 (PMC8601158; doi:10.3389/fneur.2021.753944)

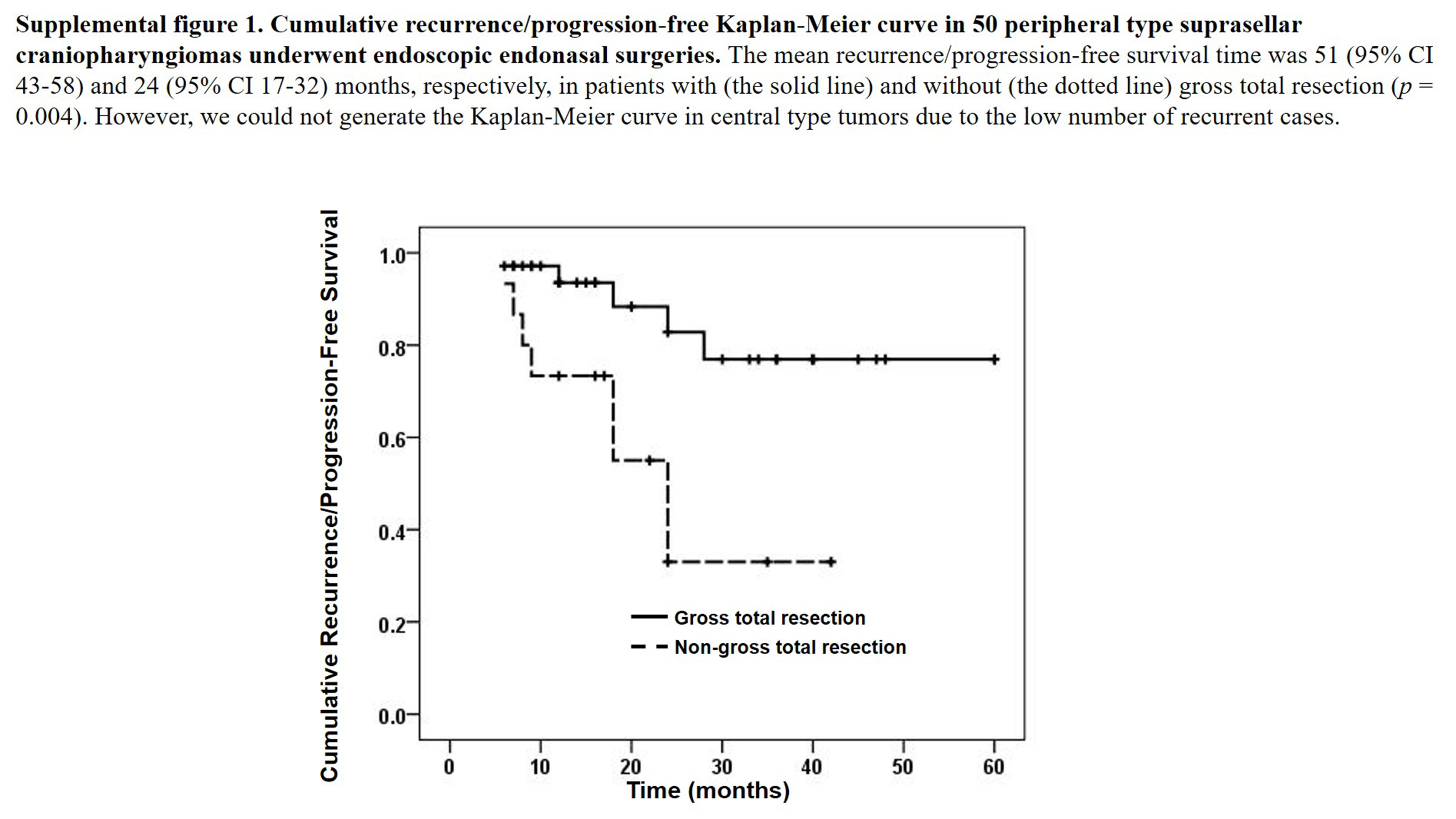

Supplement: Supplementary file 1 [file Image_1.JPEG]

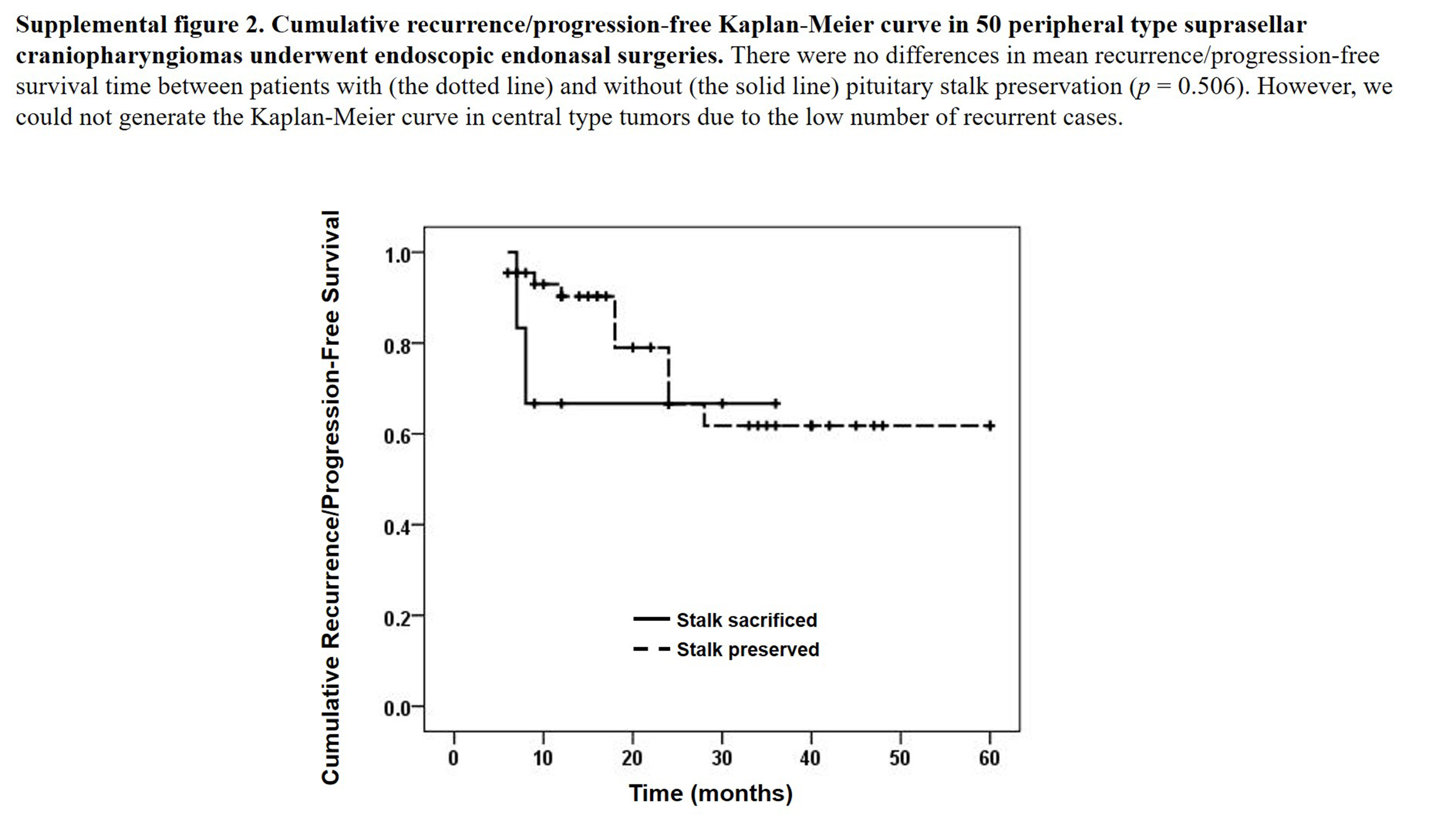

Supplement: Supplementary file 2 [file Image_2.JPEG]
